# Evaluating the effects of supplementing ward nurses on quality of newborn care in Kenyan neonatal units: protocol for a prospective workforce intervention study

**DOI:** 10.1186/s12913-022-08597-9

**Published:** 2022-10-04

**Authors:** Abdulazeez Imam, David Gathara, Jalemba Aluvaala, Michuki Maina, Mike English

**Affiliations:** 1 Health Services Unit, KEMRI-Wellcome Trust Research Programme, Nairobi, Kenya; 2 Health Systems Collaborative, Nuffield Department of Medicine, University of Oxford, Oxford, UK; 3 MARCH Centre, London School of Hygiene and Tropical Medicine, London, UK; 4 Department of Paediatrics, University of Nairobi, Nairobi, Kenya

**Keywords:** Nursing staff, Health systems strengthening, Newborn units, Human Resources for Health, Quality of care, Missed nursing care

## Abstract

**Background:**

Data from High Income Countries have now linked low nurse staff to patient ratios to poor quality patient care. Adequately staffing hospitals is however still a challenge in resource-constrained Low-middle income countries (LMICs) and poor staff-to-patient ratios are largely taken as a norm. This in part relates to limited evidence on the relationship between staffing and quality of patient care in these settings and also an absence of research on benefits that might occur from improving hospital staff numbers in LMICs. This study will determine the effect on the quality of patient care of prospectively adding extra nursing staff to newborn units in a resource constrained LMIC setting and describe the relationship between staffing and quality of care.

**Methods:**

This prospective workforce intervention study will involve a multi-method approach. We will conduct a before and after study in newborn units of 4 intervention hospitals and a single time-point comparison in 4 non-intervention hospitals to determine if there is a change in the level of missed nursing care, a process measure of the quality of patient care. We will also determine the effect of our intervention on routinely collected quality indicators using interrupted time series analysis. Using three nurse staffing metrics (Total nursing hours, nursing hours per patient day and nursing hours per patient per shift), we will describe the relationship between staffing and the quality of patient care.

**Discussion:**

There is an urgent need for the implementation of staffing policies in resource constrained LMICs that are guided by relevant contextual data. To the best of our knowledge, this is the first study to evaluate the prospective addition of nursing staff in resource-constrained care settings. Our findings are likely to provide the much-needed evidence for better staffing in these settings.

**Trial registration:**

This study was retrospectively registered in the Pan African Clinical Trial Registry (https://pactr.samrc.ac.za/Default.aspx?Logout=True) database on the 10th of June 2022 with a unique identification number-PACTR202206477083141.

**Supplementary Information:**

The online version contains supplementary material available at 10.1186/s12913-022-08597-9.

## Background

Nurses represent the largest group within the health workforce and are the backbone of health service delivery [1]. As a result, they are critical to maintaining a healthy population and for the attainment of all global development goals that relate to quality and equitable health care [1]. To underscore the important role nurses play in the global health agenda, the 74^th^ World Health Assembly designated the year 2020 as the International Year of the Nurse and the Midwife [2].

Despite being indispensable to health systems around the world, there is still a global shortage of nurses; 90% of which occur in resource-constrained LMICs resulting in extreme nursing staff-to-patient ratios that compromise the quality of patient care provided [3, 4]. These shortages cut across all sectors of the health system including the care of more vulnerable groups such as pregnant women and newborns. Human resource for health (HRH) shortages are viewed as an important factor responsible for the untimely deaths of mothers and their newborns while receiving care [5]. In addition to HRH challenges, resource-constrained settings have wider challenges with the infrastructure and material resources needed to provide quality care.

Globally, evidence on the role nurse staffing plays in ensuring the quality of patient care has largely come from High-income Countries (HICs), mainly ecological analyses of administrative health data or cross-sectional studies that are based on nurse self-report data [6–11]. These HICs have distinctly different organisational and patient care contexts to LMICs. They particularly have a better nurse to patient ratios, so there is limited evidence on nurse staffing and quality of care relationships from areas with ratios typical of LMIC contexts. The predominance of non-interventional research also limits causal inference in this research area and it is suggested that hospital-level ecological analysis might not reflect the variability occurring at lower levels of care [12, 13]. Well-designed patient-level analysis of data might provide more information on the role of nurse staffing in ensuring quality patient care, particularly in LMIC contexts where there is limited data.

Research relating nurse staffing and quality of care has largely employed outcome-based quality indicators, for example, mortality and length of hospital stay, with less work on process-based indicators that provide the link between nurse staffing and health outcomes [14]. Process-based indicators may be a more sensitive means to explore whether interventions or exposures have plausible effects. An increasingly studied process measure of quality is missed nursing care; an umbrella term that describes gaps in nursing care provision and can involve partially omitted, completely omitted or delayed clinical, emotional and administrative nursing duties [15, 16]. The study of this phenomenon has, however, largely been based on nurses self-reporting with well-known limitations of recall and social desirability bias. Ethically acceptable interventional designs that evaluate the effects on missed care based on direct observation would be an advantage.

The current study proposes to evaluate the effects on nurse-delivered care of a prospectively designed workforce intervention aimed at improving nurse staffing in select neonatal units in Kenya. The primary outcome, missed nursing care, a process-based measure of the quality of patient care will be assessed at the patient level using direct observation of care [17].

### Hypothesis

We hypothesize that adding extra ward nurses to neonatal units in Kenya will result in improvements in the process of care (decreased level of missed nursing care).

## Methods/design

### General aim

To evaluate if and how an intervention to improve neonatal ward nurse staffing levels in a resource constrained LMIC setting results in improvements in the quality of patient care provided.

### Objectives

#### Primary objective

1. To determine the effect of enhancing nurse staffing on missed nursing care measured using the nursing care index (NCI) tool in a before and after design study of four hospitals.

#### Secondary objectives

2. To examine the degree to which additional staff affects nurse staffing metrics on Kenyan newborn units, as a measure of intervention fidelity, and the relationship between these metrics and missed nursing care on these wards.

#### Pre-specified observational evaluation

3. To track changes in neonatal quality indicators derived from routinely collected data across 12 hospitals and explore differences between hospitals receiving additional staffing and those that do not.

### Research setting

This project will be introducing supplemental nurses to resource-constrained neonatal units in four hospitals aiming to offer intermediate-level neonatal care in Kenya which all belong to a Clinical Information Network (CIN). Briefly, the CIN is a learning health system that has been in existence since 2013 and spans 23 Kenyan hospitals where routine paediatric and newborn admission data are collected using structured data collection tools [18]. The information from the CIN provides feedback to the participating hospitals on the overall quality of care being provided but has also served as a platform for research on patient care quality and to assess interventions targeting better paediatric and newborn care [18]. Twelve of the 23 CIN hospitals received a bundle of neonatal technologies (phototherapy machines, Continuous Positive Airway Pressure devices, pulse oximeters and oxygen concentrators) provided by the Newborn Essential Solutions and Technology 360^o^ (NEST-360°) programme before the end of 2021 [19].

### Study design

This study will employ a prospective, multimethod approach to evaluate if an intervention to improve nurse staffing in Kenyan intermediate-level neonatal wards improves nurse-delivered care.

#### Primary and secondary objective (how supplemental nurse staffing impacts the level of missed nursing care)

**Fig. 1:**
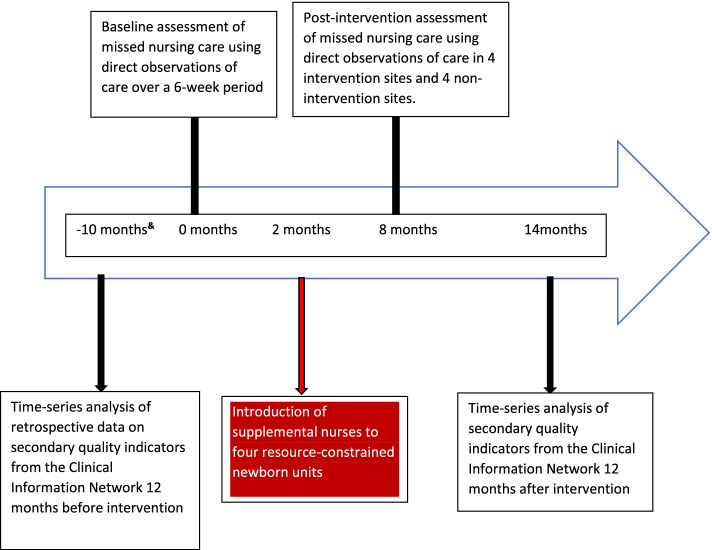
Timeline for data collection for the workforce intervention study. ^&^10 months is retrospective data collection 12 months before introducing supplemental nurses to intervention units

Using bedside observations of care, we will employ a before and after design in 4 intervention hospitals selected from the 12 NEST360 hospitals. Our baseline observations will be conducted in the 4 intervention hospitals before enhancing nurse staffing and additional observations will be performed after 6 months. At the same post-intervention time point observations will be made in a further set of 4 hospitals from the original 12 that have not received the workforce intervention (Fig. 1). Our primary analysis will be the before-and-after within-site comparison, the single time-point observations in the 4 non-intervention sites will serve as an external reference for our 4 intervention hospitals. Data on nurse staffing metrics will be collected for 6 months before the workforce enhancement and in the 6 months leading to the post-intervention assessment in the 4 intervention hospitals. These data will be used to assess whether and to what degree providing additional nurses changes nurse staffing metrics as an indication of intervention fidelity. Similar data on nurse staffing metrics will be collected from the 4 reference hospitals and then data from all 8 hospitals at the post-intervention time point will be used to address our secondary objective of exploring the relationship between nurse staffing metrics and nurse-delivered care.

#### Observational evaluation

We will use data from the 12 NEST360 hospitals (spanning the 4 intervention hospitals and 8 non-intervention hospitals) to track a set of secondarily collected quality indicators using CIN data for 12 months before and 12 months after the 4 intervention hospitals receive additional nurses. Potential intervention effects will be explored using interrupted time series analysis (Fig. 1).

### Site selection and eligibility criteria

We have selected solely public district-level health facilities that are part of the CIN and that have received a standard set of neonatal equipment and linked technical training as part of the NEST360 programme as study sites. Previous research has shown that these types of public hospitals experience the greatest level of missed nursing care associated with poor staffing levels [17]. Intervention sites are selected to be within 1 to 3 h of the Kenyan Capital, Nairobi because of travel restrictions linked to the Covid-19 pandemic enabling feasible access to these research sites. Table 1 provides a comparison of the staffing numbers and average admissions for the 4 intervention sites.

**Table 1:** Bed capacity, annual admissions, and staffing strengths across the 4 intervention hospitals

Metric	Hospital A	Hospital B	Hospital C	Hospital D
Newborn unit bed capacity^a^	53	50	38	47
Nurse staffing numbers (pre-intervention)	17	11	10	8
Expected percentage change in nurse staffing after adding 3 extra nurses (%)	17.6	27.3	30.0	37.5
**Number of other staff**				
Consultant Paediatricians	2	2	2	2
Medical officers	0	0	1	2
Medical interns	2	2	2	2
Ward clerks	3	3	1	1^b^
Average annual neonatal admissions^c^	2357	2514	1299	1800

^a^In practice, there is cot and incubator sharing and admissions out-strip the bed capacity

^b^Shared with other units

^c^2019/2020 data from a NEST-360 baseline facility assessment

#### Study population

##### Primary objective 

For our research on missed nursing care, we will recruit eligible newborn infants admitted to newborn units in selected hospitals for observations of care. We plan to recruit babies across all illness severity categories as our primary outcome, missed nursing care is associated with illness severity categories [17]. Babies for observation will be identified from previously defined categories in use in Kenyan newborn units; categories, A, B and C, where categories A are the most acutely ill babies, B are moderately ill and C are stable babies [20].

**Inclusion criteria**

1. Sick babies’ resident on the neonatal unit
2. Those whose parents/caregivers consent to the study
3. Babies on a shift where the nurse on duty has consented to observations.

**Exclusion criteria**

We will exclude the following babies:

1. Babies who are severely ill and at risk of imminent death during observations of care
2. Babies with congenital anomalies
3. Babies with surgical conditions
4. Those whose parents decline consent

##### Observational objective

For our secondary quality of care indicator analysis, our study population will be all neonates admitted to our 4 study facilities and a set of 8 non-intervention hospitals, 12 months before and 12 months after introducing supplemental newborn unit nurses. For this population, we will track the number of vital signs conducted in the first 48 h of admission and the change in newborn mortality. This data is available from the CIN database.

### Research plan

#### Intervention

The intervention to be evaluated is the addition of 3 extra nurses to selected resource-constrained newborn units for 12 months. All facilities experience very low nurse-to-patient ratios and other wider resource constraints including limited access to technology and material resources for patient care, although access to technologies has been improved by the NEST360 programme. The provision of 3 additional nurses reflects a potentially feasible nurse staffing improvement by health policymakers in a resource-challenged setting like that in Kenya and should constitute an improvement in the nurse staffing of between 17.6% and 37.5% in these units (Table 1).

#### Logic model

**Fig. 2:**
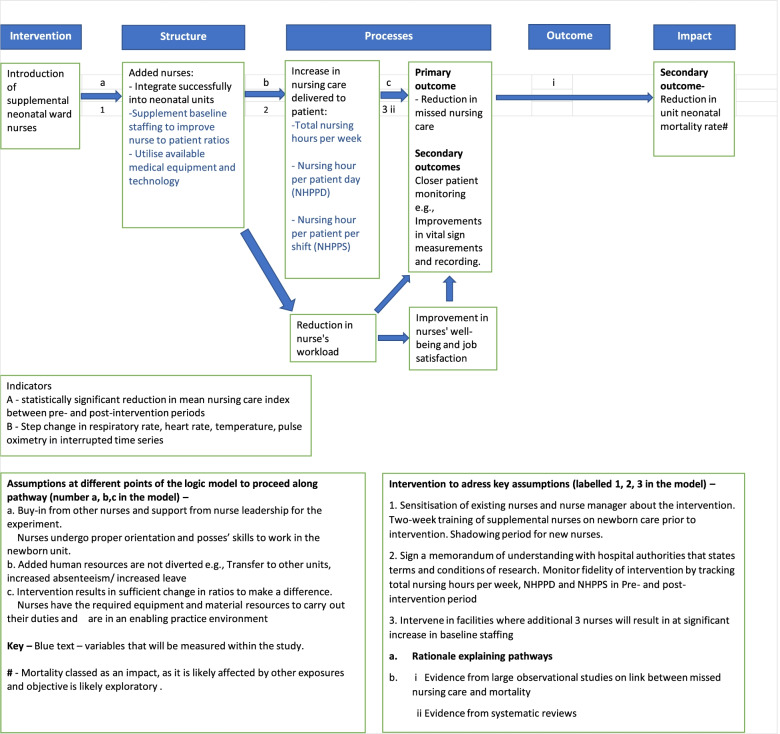
Logic model for the intervention

We developed a logic model (Fig. 2) to guide our thinking on the deployment of our intervention, identify assumptions and articulate how our intervention is likely to influence the study outcome. This model was structured around the Donabedian structure-process-outcome framework where health system structures are likely to influence processes which in turn influence health outcomes [21]. Our intervention changes a key element of structure, nurse staffing, and is likely to affect nurse-delivered care and subsequently neonatal outcomes through our hypothesised pathway (Fig. 2). Key assumptions that along the proposed causal pathway for our intervention to have the desired effect include: i) support for the new nurses from the existing nursing staff, ii) availability of equipment and other routine resources for nurses to conduct their activities, and iii) support from the hospital authorities and nursing service manager such that additional human resources are not diverted to other understaffed parts of the hospital. To help address the last concern we sought agreements that hospitals would not divert human resources from the NBU to other hospital units following the addition of supplemental nurses and that they might replace any nurses who leave the unit. Additionally, the supplemental nurses will be trained in the provision of essential newborn care using approaches developed by the NEST360 programme and offered to existing hospital staff. To support the intervention a memorandum of understanding with the county governments of our intervention hospitals was signed.

#### Intervention fidelity

From our logic model, we identified 3 key mediating variables that are useful measures of intervention fidelity i.e., variables that measure the magnitude or intensity of our intervention (Fig. 2). These variables are process variables and measure the time available for delivering nursing care. These metrics include:

- The total number of nursing hours per month assessed as the cumulative total number of nursing hours delivered per month
- Nursing hours provided per patient day assessed as the total nursing care hours provided to patients in a month/ Total number of patients admitted during the same defined period
- Nursing hour per patient per shift which is the total registered nursing shift hours divided by the total number of patients at the beginning of a shift.

If the intervention is deployed in the proposed manner, all three variables are likely to increase post-intervention with the first two metrics assessing broad changes in staffing and the third focused on specific periods including those that are subject to direct observation.

#### Outcomes

##### Primary outcome

For our primary objective (how supplemental nurse staffing impacts the level of missed nursing care), our outcome will be the change in the magnitude of missed nursing care, which will be measured as a change in a Nursing Care Index (NCI). The NCI is an unweighted aggregate patient-level score of all the items of care that are delivered to a baby compared with all the items of expected care [17]. A lower NCI score means more care is missed while a higher score means more care is carried out by nurses for the baby.

Missed nursing care is a process measure of the quality of care provided and it might be more sensitive to a change in nursing numbers [14]. Additionally, it is a direct marker of the nursing process and is an early precursor to adverse patient health outcomes [14].

##### Observational outcome

Data on the recording of vital signs by nurses (respiratory rate, heart rate, temperature, and pulse oximetry) is routinely collected as part of CIN activities. Prior research and unpublished observations show it is uncommon for these observations to be taken and recorded at the recommended minimum frequency of every 6 h for the first 48 h of admission, a problem at least in part attributed to poor nurse-to-patient ratios [22, 23]. An improvement in vital signs monitoring temporally associated with the intervention of enhancing nurse staffing would provide support for the hypothesis that better staffing improves care quality. Mortality is a distal outcome in our hypothesized causal pathway (Fig. 2) as it is likely to be affected by multiple influences (e.g. case-mix) [24]. Changes in mortality a measure of the effectiveness of our intervention and as an important outcome for policymakers will be examined in exploratory analysis.

#### Data collection tool

##### Missed nursing care data collection tool

To measure the level of missed nursing care before and after our intervention (our primary objective), we will employ an adapted version of the Nursing Care Index (NCI) tool [17] (Additional file 1). This tool involves performing non-participatory observations of nursing care provided to newborns using a structured checklist (supplementary material). Our adaptation to the existing tool includes key data on potential confounders of the relationship between nurse staffing and missed nursing care, for example, proxy data to estimate nursing workload on shifts (number of high acuity babies, babies on interventions such as Continuous Positive Airway Pressure), support staff available on shift, and presence of the necessary medical equipment for carrying out certain nursing duties such as pulse oximetry recordings. We will pilot our adaptation of the NCI tool for feasibility.

#### Observations of care

We will employ four observers across our 4 intervention sites to conduct bedside observations of care. These will be staff with a qualification in Allied Health Sciences, for example, nutrition science as proved successful in prior work [17]. We will avoid employing nurses or medical doctors as this group are likely to ascribe their professional standards to the care they are observing, and it would be challenging for them to retain non-participant status.

**Fig. 3:**
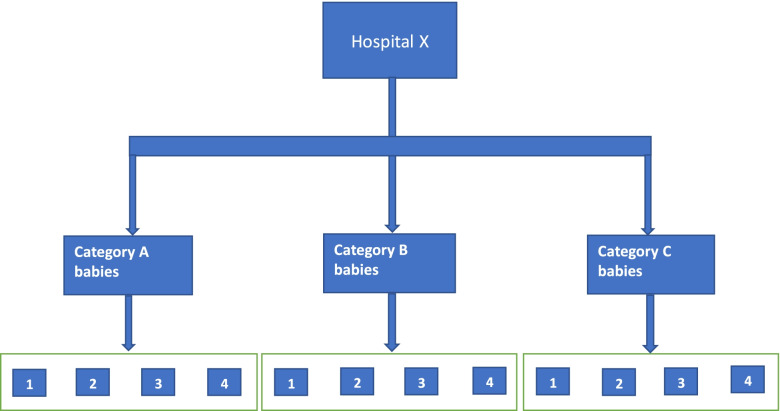
Sampling approach across hospital facilities. 1 – Random Weekend Day 12-h shift, 2—Random Weekend night 12-h shift, 3- Random Weekday Day 12-h shift, 4- Random Weekday night 12-h shift. Weekend and weekday shifts are in a 5:3 ratio

Because missed nursing care has previously been shown to vary across shift timing (day and night, weekday and weekend) and also patient severity [17], we will employ stratified data collection with the aim to equally distribute observations across the morning and night shifts and in a 5: 3 ratio across weekdays and weekends. This is carried out to maximise variation in the observational periods and to have a representative estimate of missed care across all shifts. Our stratified data collection will also involve conducting observations on babies with different illness severities (Fig. 3).

#### Statistical power

Drawing on prior experience for our primary outcome [17], assuming a 5% improvement in mean NCI scores (SD- 15%), adjusting for clustering with a design effect of 1.5 and setting α and β at 5% and 80% respectively, the minimum sample size for this study will be 213 in each period (pre-intervention and post-intervention). This will be split equally across the 4 hospitals (55 babies in each hospital per period). This will further be split across randomly sampled 12-h weekday and weekend shifts and across illness severity categories (A, B, C) (Fig. 3). In each shift, we will aim to observe 4 co-located babies for the entire duration of the randomly sampled 12-h blocks. This has been deemed logistically feasible based on prior experience using the NCI tool within the same setting [17].

### Patient and public involvement

There was no patient or public involvement in the design and conduct of this study.

### Statistical analysis

We will use summary statistics to describe our study's contextual data (e.g., monthly admission numbers, and patterns of diagnosis). To determine if there is a change in the level of missed nursing care across periods (pre-and post-intervention), we will use linear regression analysis with the NCI as the outcome variable and observation period as the main independent variable. Linear regression will also be used to assess the effect of intervention after adjusting for potential confounders such as patient severity category, and observation made on a weekend/weekday. We will report the risk ratios and corresponding 95% confidence intervals. For our secondary objectives, we will use interrupted time series analysis to track changes in the secondary quality indicators.

### Dissemination plan

We intend to present the result of this study to the participating hospitals, relevant stakeholders including the Ministry of Health and professional associations, at conferences and publish them in scientific peer-reviewed journals.

### Confidentiality

Data collected using the study observational checklist (supplementary material) will be kept securely at the KEMRI Wellcome Trust Research Programme (KWTRP) and entered on-site into password-protected computers with encryption using Redcap software. Data from individual deidentified observational checklists will be collated on a central server maintained by the KWTRP after being encrypted for transmission over the internet. De-identified data shared with KWTRP will be stored in secure servers with password-protected access and as guided by KEMRI – Wellcome Trust ICT and Data Management policies. Data held on these servers are backed up in similar password protected mirror servers at another location within the KEMRI – Wellcome Trust.

## Discussion

Both material and human resources are crucial to maintaining the quality of patient care. Interventions to improve in-patient quality of care in resource-constrained settings have largely ignored a critical element for quality service delivery which is having an adequate number of health staff. For example, many interventions focus on enhancing technology or skill acquisition of existing local health workers. Yet, these require the presence of a sufficient numbers of staff to promote optimal effectiveness and ensure patient safety.

To the best of our knowledge, this is the first prospective workforce intervention study that proposes to supplement nurses within a hospital setting to examine the association between improved staffing and quality of care. Previous research has been retrospective, evaluating the effects of improvements in staffing in the form of natural experiments or has involved prospective supplementation of nursing assistants [25–27].

We recognise that the Hawthorne effect is a limitation of studies that involve direct observations [28], and we plan to minimise the effect of this on our data collection. Similar to our earlier work using the NCI tool, before the current data collection, we will conduct training of the observers and have a one-week familiarisation period within our study sites [17]. This period involves observers learning the layout and routines of each unit and this also serves to make the nurses within the units comfortable with the observer. We will spread our data collection across randomly selected 12-h nursing shifts over 5 to 6 weeks that are not known to the nursing staff in advance while evidence also suggests that following the continuous presence of an observer for longer periods, the Hawthorne effect is likely to diminish [29].

## Supplementary Information

**Additional file 1.**

**Additional file 2.**

## Data Availability

Not applicable.

## Abbreviations

CIN: Clinical Information Network
HIC: High Income Country
HRH: Human Resources for Health
NCI: Nursing Care Index
NEST-360^o^: Newborn Essential Solutions and Technology 360^o^
LMIC: Low-middle Income Country
