# Supplementary material for: Evaluating the effects of supplementing ward nurses on quality of newborn care in Kenyan neonatal units: protocol for a prospective workforce intervention study
Source: BMC Health Serv Res. 2022 Oct 4;22:1230. doi: 10.1186/s12913-022-08597-9 (PMC9530438; doi:10.1186/s12913-022-08597-9)
Supplement: Supplementary file 1 — Additional file 1. [file 12913_2022_8597_MOESM1_ESM.pdf]

CIN Hospital code |__|__| Subject ID |__|__|

Neonatal nursing care observational checklist

Date of completion	|__|__|/|__|__|/|__|__|__|__| **(DD/MM/YYYY)**	
Observer code	|__|__| (e.g., 01)	
**Subject ID**	|__|__|- |__|__| (4-digit code (first 2 digits, hospital code and last 2-digit, patient number e.g., 10)	In-patient number

**Part A – Shift information (Tick as appropriate)**			
Q 1. Shift type			
Weekday day	☐	Weekday night	☐
Weekend day	☐	Weekend night	☐
Q 2. Observation period			
Start Date<br>|__|__|/|__|__|/|__|__|__|__| **(DD/MM/YYYY)**		Start time<br>|__|__| :|__|__| (Please use 24-hour timing e.g., 23:59, not 11:59pm)	
End Date<br>|__|__|/|__|__|/|__|__|__|__| **(DD/MM/YYYY)**		End time<br>|__|__| :|__|__| (Please use 24-hour timing e.g., 23:59, not 11:59pm)	

Q 3. Total number of patients in the ward (Include all babies including those in KMC and acute rooms)	|__|__|__|	Number of babies on CPAP	|__|__|
		Number of babies on Oxygen	|__|__|
		Number of babies on IV therapy (medications/ fluids)	|__|__|
		Number of babies on Phototherapy	|__|__|
		Number of babies requiring nasogastric tube/orogastric feeding	|__|__|
		Number of babies requiring incubator care	|__|__|
Q 4 Is there an extra KMC Ward/ room managed by the NBU nurses?	Yes ☐<br>No ☐	Number of babies on the KMC ward<br>|__|__|	

CIN Hospital code |__|__| Subject ID |__|__|

Q 5 Is there an acute room present in the NBU?	Yes ☐<br>No ☐		How many babies are present in this room?<br>|__|__|		
Q6. Number of nurses on current 12-hour shift	Actual (Based on observations)				
	Morning shift (07:30 – 12:30)	☐	Night shift (18:30 – 07:30)	☐	
**Comment:**	Evening shift (07:30 – 16:30)	☐	Others __________	☐	
	Afternoon shift (12:30 – 18:30)	☐			

Q 7. Device check. On the current shift, how many of the following devices below are present and functional					
Glucometer **(If no strips, put as 0)**	☐	Weighing scale	☐	Pulse oximeter	☐
Thermometer **(owned by the unit)**	☐	Stethoscopes **(owned by the unit)**	☐		

Instruction: Please reconcile the following information below at end of the shift

Q 8. How many of the following processes occurred during the shift?	Admissions	|___|
	Discharges	|___|
	Referrals	|___|
	Deaths	|___|
Q 9. How many of the following staff cadres where present on this shift?	Medical officer	|___|
	Clinical Officer Interns	|___|
	Medical officer interns	|___|
	Nursing officer interns	|___|
	Nursing students	|___|
	Others ____________	|___|
	Others ____________	|___|
Q 10. Was the ward-in-charge or deputy ward-in-charge present at any time during the 12-hour observation period	Yes ☐	

CIN Hospital code |__|__| Subject ID |__|__|

	No ☐	
**Part B – Baby's biodata (Tick as appropriate)**		
Q 11. Care category		
Category A (Critical/HDU) ☐	Category B (Acute) ☐	Category C (Stable) ☐
Q 12. Date of admission<br><br>|__|__|/|__|__|/|__|__|__|__| **(DD/MM/YYYY)**		
Q 13. Current age (write in days if above 24 hours old, if less than please write in hours)	Hours |__|__| Days |__|__|	
Q14. Gender		
Male ☐ Female ☐		
Q 15. Current diagnosis	Diagnosis 1 (Primary diagnosis)<br>_______________	
	Diagnosis 2 (Secondary diagnosis)<br>_______________	
	Diagnosis 3 (Secondary diagnosis)<br>_______________	
	Other diagnosis<br>_______________	
Q 16. Birth weight (grams)<br><br>|__|__|__|__|		
Q 17. Current weight (Most recent weight in grams)<br><br>|__|__|__|__|		

CIN Hospital code |__|__| Subject ID |__|__|

Nursing tasks	Task frequency	Expected number within current 12-hour shift	Tasks done (Insert Y, if yes and N if no)	Time tasks done	Comment
**Routine nursing care**					
Nurses handing over patient		1	☐	☐	
Patient assessment before shift		1	☐	☐	
Nurse washes / sanitises hands before patient assessment (Assess the first patient contact)		1	☐	☐	
Nurse attends the ward round with doctor (s) to see patient. (If nurse student, mark as no)		1	☐	☐	
Nurse communicates with caregiver details of care/ counsels the caregiver		1	☐	☐	

CIN Hospital code |__|__| Subject ID |__|__|

**Nursing tasks**	**Task frequency (Circle option)**	**Expected number within current 12-hour shift**	**Tasks done (Insert Y, if yes and N if no)**	**Task performed by (N for nurse, NS* –Student nurse, M-Caregiver/ Mother, W-ward clerk)**	**Time tasks done**	**Comment**
Temperature check	4/6/12 hourly	1 /2 /3	☐	☐	☐	
			☐	☐	☐	
			☐	☐	☐	
Pulse/heart rate check	4/6/12 hourly	1 /2 /3	☐	☐	☐	
			☐	☐	☐	
			☐	☐	☐	
Respiratory rate	4/6/12 hourly	1/ 2 /3	☐	☐	☐	
			☐	☐	☐	
			☐	☐	☐	
Pulse oximetry	4/6/12 hourly	1/ 2 /3	☐	☐	☐	
			☐	☐	☐	
			☐	☐	☐	

*- Task performed by a nursing student under supervision should be termed as done by a nurse

CIN Hospital code |__|__| Subject ID |__|__|

**Nursing tasks** (routine newborn care)	**Task frequency (Circle option)**	**Expected number within current 12-hour shift**	**Tasks done (Insert Y, if yes and N if no)**	**Task performed by (N for nurse, NS* –Student nurse, M-Caregiver/ Mother, W-ward clerk)**	**Time tasks done**	**Comment**
Cleaning baby	[redacted]	1			[redacted]	
Linen change	[redacted]	1			[redacted]	
Weight check	[redacted]	1			[redacted]	
Checking incubator settings	[redacted]	1			[redacted]	
Diaper change	[redacted]	1			[redacted]	
Cord care	[redacted]	1			[redacted]	
Turning the baby	[redacted]	4			[redacted]	

*- Task performed by a nursing student under supervision should be termed as done by a nurse

CIN Hospital code |__|__| Subject ID |__|__|

**Nursing tasks**<br><br>**(Feeding)**	**Task frequency (Circle option)**	**Expected number within current 12-hour shift**	**Tasks done (Insert Y, if yes and N if no)**	**Task performed by**<br>**(N for nurse, NS* –Student nurse, M-Caregiver/ Mother, W- ward clerk)**	**Time tasks done**	**Comment**
Breastfeeding ☐<br>Cup/spoon feeding ☐<br>Nasogastric/ Oro-gastric tube ☐<br>No oral feeding ☐	3-hourly	4	☐	☐	☐	
			☐	☐	☐	
			☐	☐	☐	
			☐	☐	☐	
**NG Tube feeding process &**						
Check tube positioning	3-hourly	4	☐	☐		
			☐	☐		
			☐	☐		
			☐	☐		
Measure feeds	3-hourly	4	☐	☐		
			☐	☐		
			☐	☐		
			☐	☐		

Position the baby after feeding	3-hourly	4	☐	☐		
			☐	☐		
			☐	☐		
			☐	☐		

& - Complete for only NG-tube fed babies

CIN Hospital code |__|__| Subject ID |__|__|

Nursing tasks (Medication)	Task frequency (Please circle)	Expected number within current 12-hour shift	Tasks done (Insert Y, if yes and N if no)	Time tasks done		Comment
**IV Medication**						
Drug name ____________________________	6/8/12/24 hourly	|___|	☐	☐		
			☐	☐		
Drug name ____________________________	6/8/12/24 hourly	|___|	☐	☐		
			☐	☐		
Drug name ____________________________	6/8/12/24 hourly	|___|	☐	☐		
			☐	☐		
Drug name ____________________________	6/8/12/24 hourly	|___|	☐	☐		
			☐	☐		
6 hourly – QDS/QID, 8 hourly – TDS/TID, 12 hourly – BD, 24 hourly - OD						

CIN Hospital code |__|__| Subject ID |__|__|

**Nursing tasks (Medication)**	**Task frequency**	**Expected number within current 12-hour shift**	**Tasks done (Insert Y, if yes and N if no, E if not determined)**	**Time tasks done**	**Task performed by (N for nurse, NS* –Student nurse, M-Caregiver/ Mother, W-ward clerk)**	**Comment**
**Oral Medication**						
Drug name ____________________________	12/24 hourly	|___|	☐	☐	☐	
Drug name ____________________________	12/24 hourly	|___|	☐	☐	☐	
Drug name ____________________________	12/24 hourly	|___|	☐	☐	☐	
Drug name ____________________________	12/24 hourly	|___|	☐	☐	☐	
**IV Medication delivery process (Perform this observation for first round of medications)**						
Review of treatment sheet			☐			
Cannula flush with saline before drug administration			☐			
Cannula flush with saline after drug administration			☐			

CIN Hospital code |__|__| Subject ID |__|__|

Nursing tasks (Special newborn care)	Task frequency	Expected number within current 12-hour shift	Tasks done (Y-yes, N-no)	Time tasks done	Comment
**Phototherapy**					
Turning/positioning	4 hourly	3	☐<br>☐<br>☐		
Skin assessment	6 hourly	2	☐<br>☐		
Eye care	12 hourly	1	☐		
Changing eye pads	12 hourly	1	☐		
**Continuous Positive Airway Pressure**					
Checking nasal prong position	4 hourly	3	☐<br>☐<br>☐		
Checking oxygen flow rate	4 hourly	3	☐		

			☐<br>☐		
**Kangaroo Mother Care**					
Supervision of mother during KMC			☐		

CIN Hospital code |__|__| Subject ID |__|__|

**Documentation of tasks (source)**	**Not Done (Please tick)**	**Done (Please tick)**	**If done, how many times (Please write number)**	**Comments**
Neonatal assessment by nurse (Nursing cardex)	☐	☐		
Nursing care plan (Nursing cardex/ care plan)	☐	☐		
Temperature measurements	☐	☐	☐	
Heart rate	☐	☐	☐	
Respiratory rate	☐	☐	☐	
Oxygen saturation	☐	☐	☐	
Ward round details	☐	☐		
Frequency and volume of feed	☐	☐	☐	
Health talks/ Communication to parents	☐	☐	☐	
Volume of IV fluids	☐	☐	☐	
Weight check	☐	☐	☐	
