# Supplementary material for: Evaluating the effects of supplementing ward nurses on quality of newborn care in Kenyan neonatal units: protocol for a prospective workforce intervention study
Source: BMC Health Serv Res. 2022 Oct 4;22:1230. doi: 10.1186/s12913-022-08597-9 (PMC9530438; doi:10.1186/s12913-022-08597-9)
Supplement: Supplementary file 2 — Additional file 2. [file 12913_2022_8597_MOESM2_ESM.pdf]

Staff and Mothers observation information and consent

HIGH Q Study Form 2k - <u>Information Sheet for gaining written informed consent from hospital staff and mothers to observe newborn ward routines</u>

Title: Evaluating the effects of technology and workforce enhancement to support neonatal hospital care in Kenya.

Lay Title: A study to learn if and how the introduction of new technologies and service delivery innovations improves the quality of care provided in newborn units

Institution	Investigators and Collaborators
KEMRI Wellcome Trust Research Programme	Dr. Michuki Maina, Dr. Dorothy Oluoch, Dr. David Gathara, Prof. Mike English, Prof. Sassy Molyneux, Prof. Caroline Jones, Joyline Jepkosgei, Dr. Jalemba Aluvaala, Dr. Tim Tuti, Ms. Edith Gicheha, Mr. John Wainaina, Mr Livingstone Mumelo, Peris Musitia, Edna Mutua.
University of Nairobi	Dr Jalemba Aluvaala, Prof Grace Irimu, Prof Fred Were, Dr Brian Maugo
Ministry of Health	Dr Caroline Mwangi, Dr Laura Oyiengo
Moi University	Prof Fabian Esamai
Nursing Association of Kenya	Edna Talam
Neonatal Nurses Chapter	Josephine Bariu
Preemie Love Foundation	Ruby Kimondo
Nuffield Department of Medicine, University of Oxford, UK	Dr. Chrysanthi Papoutsi, Dr Chris Paton, Dr. Lisa Hinton, Ms. Gulraj Grewal, Dr. Abdulazeez Imam, Dr. Gloria Ngaiza, Ms. Naima Nasir, Dr Sebastian Fuller, Dr Jacob Mc Knight, Ms. Asma Rababeh, Ms. Christiane Hagel

You are being asked to take part in a study. The box below tells you important things you should think about before deciding to join the study. We will provide more detailed information below the box. Please ask questions about any of the information before you decide to participate. You may also wish to talk to others (for example, your line manager, family, or friends) about this study, before agreeing to join.

Key Information for You to Consider
• **Voluntary Consent**. You are being asked to volunteer for a research study. You can choose whether you would like to participate or not. If you do agree, you can change your mind at any time and withdraw from the research. This will not affect you now or in the future.<br>• **Purpose**. We are doing this research to find better ways of integrating necessary technologies into Kenyan health systems. In this study, we want to understand how the introduction of new

technologies affect how nurses go around their work of caring for the newborns and if increasing the nursing workforce improves how the care is provided. Additionally, we aim to better understand if family experiences as their babies are treated will improve the acceptability of these technologies and improve patient care and follow up. We also aim to understand the governance structures that assess the safety and authorize the introduction of new technologies in the county

- **Duration.** Your participation in this study will last the length of your shift.
- **Procedures and Activities.** We will observe as you carry out your nursing tasks during your shift
- **Risks or disadvantages.** There are no known harms/disadvantages in this study that could happen to you if you join. In this study, there is a possibility that you might be a little bit inconvenienced by the time it takes to complete the interview.
- **Benefits**. There are no direct benefits in this study. However, the outputs from this study will contribute to the improvement of hospital care for newborns. If the interventions are successful, the strategies that we are testing could be adopted much more widely across Kenya.

Who is carrying out this study, and what is this study about?

This study is being carried out by KEMRI in collaboration with the University of Oxford, University of Nairobi, Moi University, Nursing Association of Kenya, Neonatal Nurses' Chapter, Preemie Love Foundation and the Kenya Paediatric Association. KEMRI is a Kenyan government organization that carries out medical research to find better ways of preventing and treating illness in the future for everybody's benefit.

In this research, we want to learn more about how to help hospitals provide better care for the sick babies that need to stay in the hospital for treatment soon after they are born. We are doing this by working with four hospitals. As part of this work, we will introduce existing technologies; educate health care providers; and provide supportive supervision and mentorship. We will assess the implementation process and the effects of these technologies on the care provision process and quality of newborn care. We will use this information to better understand to promote improvements in care and better design intervention implementation activities. To do this, we would like to observe health workers as they take care of the babies in the NBU and MCH in the course of their shifts.

Where is the study taking place, how many people does it involve, and how are they selected?

The study is taking place in 4 hospitals in Kenya. We try and make non-participatory observations for periods of several hours at a time, sometimes at the night and weekends too, over a period of 5 to 6 weeks so we can get as complete a picture as possible of newborn care in this hospital. Our observations could involve all the staff on duty on the days and at the times we come to the ward – we do not plan to observe any single person.

What does non-participatory observation as part of the study involve for those who are in it?

The person who is the observer is just there to watch and learn. Before starting any observations, the observer explains the project (as I am doing now) and asks permission of those who are on the ward if it is OK to be there. They may make notes of what they see to remind them later of the way people work but these notes will never have anyone's name in them. The person observing is **not** there to help with

giving care and will make every effort not to inconvenience people. At any time, the staff or families can ask the observer to leave the ward and this will not cause any problem.

Are there any risks or disadvantages to me / my child of taking part?

- The observations will take place during some of the nursing shifts.
- We do not believe there are any risks to your taking part as we aim to ensure the confidentiality of all participants so that no comments can be directly linked to any person.

Are there any advantages to me taking part?

There are no individual benefits to taking part. In permitting these observations to occur, you will contribute to knowledge on how to introduce technologies in newborn units as part of efforts to improve care on newborn units in Kenyan hospitals can work best. This may help other people in Kenya and elsewhere in the future, for example, through making the current approaches to improvement better or developing new strategies to improve care.

Who will have access to the information I give?

- All of our documents/ recordings are stored securely in locked cabinets and on password-protected computers. The knowledge gained from this research will be shared in summary form, without revealing individuals' identities, with study participants, hospital managers, policymakers and professional regulatory bodies.
- In future, information collected or generated during this study may be used to support new research by other researchers in Kenya or other countries on improving newborn service delivery. In all cases, we will only share information with other researchers in ways that do not reveal individual participants' identities. For example, we will remove such as their names and where they live and replace this information with number codes. Any future research using information from this study must first be approved by a local or national expert committee to make sure that the interests of participants and their communities are protected.
- The University of Oxford is responsible for ensuring that Oxford staff involved in the study in Kenya adhere to the safe and proper use of any personal information you provide, solely for research purposes.

Who has allowed this research to take place?

All research at KEMRI has to be approved before it begins by its Scientific and Ethical Review Unit (SERU) who look carefully at planned work. They must agree that the research is important, relevant to Kenya and follows nationally and internationally agreed research guidelines. This includes ensuring that all participants' safety and rights are respected.

What will happen if I refuse to participate?

All participation in research is voluntary. You are free to decide if you want to take part or not. If you do agree, you can change your mind at any time without any consequences.

What if I have any questions?

You are free to ask me any question about this research. If you have any further questions about the study, you are free to contact the research team using the contacts below:

Dr. Michuki Maina, KEMRI Wellcome Trust Research Programme, P.O. Box 43640, Nairobi 00100. Telephone: 0722248890

If you want to ask someone independent anything about this research, please contact:

Community Liaison Manager, KEMRI Wellcome Trust Research Programme, P.O. Box 230, Kilifi. Telephone: 041 7522 063, Mobile 0723 342 780 or 0705 154 386

And

The Head, KEMRI Scientific and Ethics Review Unit, P. O. Box 54840-00200, Nairobi; Telephone numbers: 0717 719477; 0776 399979 Email address: seru@kemri.org

KEMRI-Wellcome Trust Research Programme consent form for a study to learn if and how the introduction of new technologies and service delivery innovations improves the quality of care provided in newborn units

I have had the study explained to me. I have understood all that has been read/explained and had my questions answered satisfactorily. And I agree to take part in this research

I agree for the observation to take place ☐ Yes ☐ No

I understand that I can change my mind at any stage, and it will not affect me in any way.

Signature: ________________________________ **Date:** ________________

Participant/parent/guardian ________________________________ **Time:** ______________

(Please print name)

Where participant cannot read, a witness* may observe the consent process and sign below if needed:

I attest that the information concerning this research was accurately explained to and apparently understood by the participant and that informed consent was freely given by the participant.

Witness' signature: ____________________________________ **Date** _____________

Witness' name: ____________________________________ **Time** ______________

(Please print name)

**A witness is a person who is independent from the study or a member of staff who was not involved in gaining the consent.*

Thumbprint of the participant as named above if they cannot write:

I have followed the study procedure to obtain consent from the participant. S/he apparently understood the nature and the purpose of the study and consents to the participation in the study. S/he has been given an opportunity to ask questions which have been answered satisfactorily.

Designee/investigator's signature: ____________________________ **Date** ____________

Designee/investigator's name: ____________________________**Time** ____________

(Please print name)

THE PARTICIPANT SHOULD NOW BE GIVEN A SIGNED COPY TO KEEP
